# Telomere fusions as a signal of term placental aging? A pilot study

**DOI:** 10.1530/RAF-22-0065

**Published:** 2022-11-14

**Authors:** Fabiana B Kohlrausch, Fang Wang, Danxia Luo, Rebecca Mahn, David L Keefe

**Affiliations:** 1 Departamento de Biologia Geral, Universidade Federal Fluminense, Niterói, Rio de Janeiro, Brasil; 2 Department of Obstetrics and Gynecology, New York University, Langone Medical Center, New York, New York, USA

**Keywords:** placenta, hESC, genomic instability, telomere, fusion

## Abstract

The placenta plays an essential role at the beginning of life, nourishing and supporting the fetus, but its life span is limited. In late pregnancy, the placenta develops signs of aging, including inflammation and impaired function, which may complicate pregnancy. Placentas also show another sign of aging – cells with extra or missing chromosomes. Chromosomally abnormal cells could gather in the placenta if they get stranded there and/or if the cells do not separate normally. Chromosome separation goes wrong in aging cells when the DNA sequences, which protect the ends of the chromosomes, erode. When chromosomes lose their protective caps, they fuse which leads to abnormal numbers of chromosomes. In this pilot study, for the first time, we found fusions between the caps in a human placenta when it reaches full term. More studies are needed to decide whether this has an influence on how the placenta works and outcomes of pregnancy.

The placenta is the principal organ of gas and nutrient exchange between mother and fetus. The duration of pregnancy in humans is tightly controlled, and cellular senescence may participate in this timing (Cox & Redman 2017).

Telomeres are the terminal ends of linear chromosomes, which maintain genome integrity and proper chromosome segregation. Free chromosome ends expose DNA to double-strand break repair, producing telomere fusions and triggering a cell crisis, characterized by arrest, genome instability and cell death, contributing to aging (Cleal *et al.* 2018).

To examine telomere fusions, 16 human term placentas (38–41 weeks gestation) were collected from anonymous donors delivering at NYU Langone Health. Random pieces of 25–30 mg were excised from the four quadrants. NIH-approved human embryonic stem cells (hESCs) were obtained from Rockefeller University (RUES2) and University of Wisconsin (WA26). This study was approved by the NYU Langone IRB and ESCRO. Genomic DNA was amplified by polymerase chain reaction (PCR) using primers targeting subtelomeric regions, followed by nested PCR (N-qPCR) using a locked nucleic acid probe [TTAGGG]_4_ (Hata *et al.* 2018). All first N-qPCR products were examined using electrophoresis to confirm the presence (fusion positive) or absence (fusion negative) of amplicons.

All placenta samples exhibited signals consistent with telomere fusions involving one or more autosomes (Fig. 1A) and two samples involving Xp and Yp (Fig. 1B). No evidence of telomere fusions was observed in any passage of hESC lines after the first (Fig. 1C) or second N-qPCR (Fig. 1D). WA26 passage 28 exhibited a very low signal in the first N-qPCR (Fig. 1C) and a faint band around 500 bp in electrophoresis (Fig. 1E), but the second N-qPCR was consistent with no fusion signals (Fig. 1D). Electrophoresis confirmed the absence of fusions for the remaining samples (Fig. 1E).

**Figure 1:**
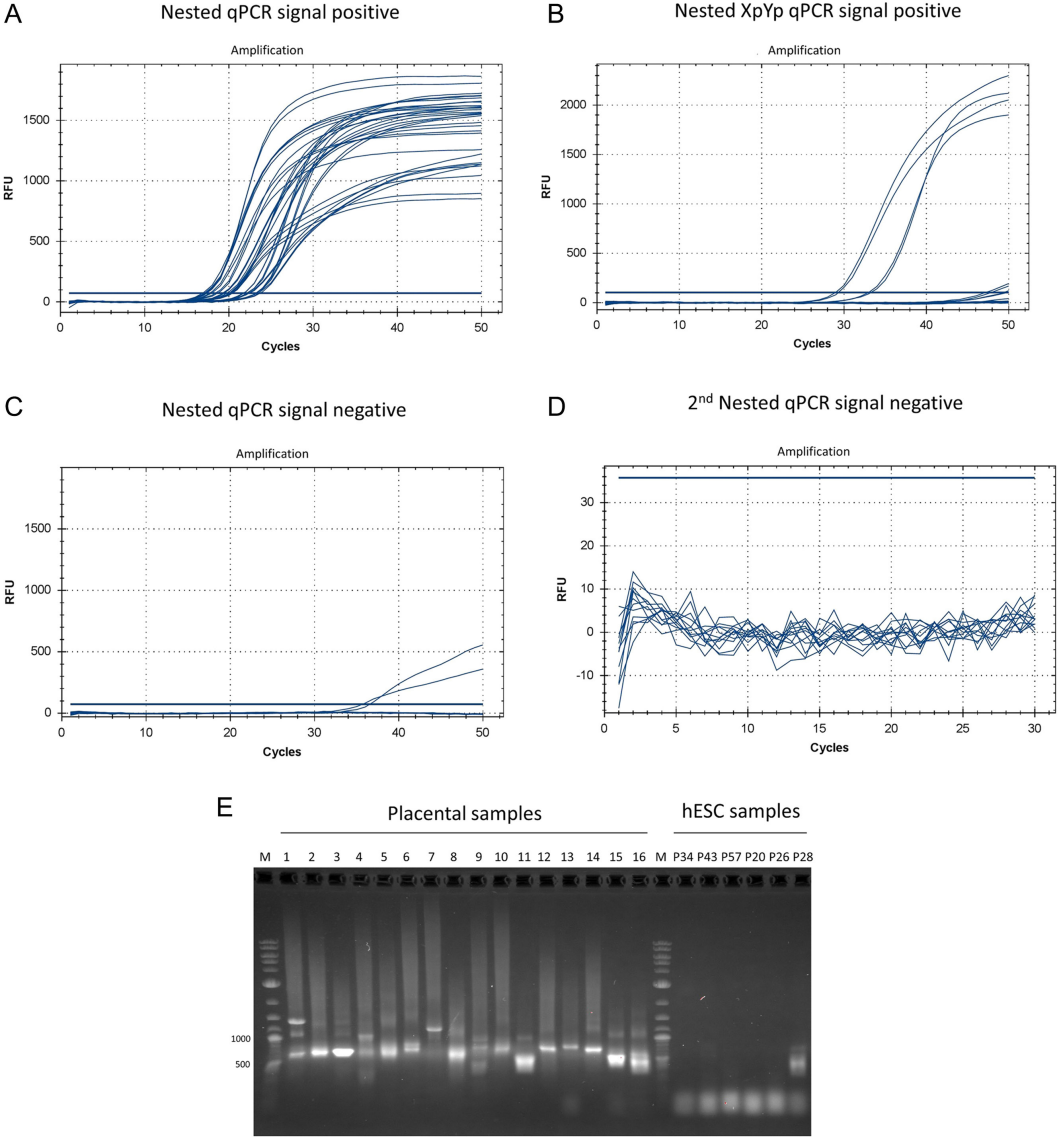
Results of telomere fusion assay in human placenta samples and hESCs. (A) Signal positive curves of placenta samples after the first nested real-time quantitative PCR (qPCR), using the locked nucleic acid probe targeting [TTAGGG]_4_ and diluted first PCR products. All samples showed a Ct below 35. (B) Signal positive curves after XpYp first nested real-time qPCR, using the locked nucleic acid probe targeting [TTAGGG]_4_ and diluted first PCR products. (C) Signal negative curves in both hESC lines after the first nested real-time quantitative PCR (qPCR), using the locked nucleic acid probe targeting [TTAGGG]_4_ and diluted first PCR products. (D) Signal negative curves after the second nested real-time qPCR in hESCs using the locked nucleic acid probe targeting [TTAGGG]_4_ and diluted first nested-qPCR products. (E) Gel imaging of signal positive and negative first nested-qPCR products. All samples were submitted to the second nested real-time qPCR in hESCs using the locked nucleic acid probe targeting [TTAGGG]_4_ and showed no positive signal of fusion (see D). Lanes 1–16: positive telomere fusions in placenta samples, Lanes P34, P43, P57 (RuES), P20, P26 and P28 (WA26): negative telomere fusions in hESC samples from different passages. All samples were run in duplicate.

Fetoplacental membranes and maternal decidua display features of senescence as pregnancy approaches term (Cox & Redman 2017). The reported presence of telomere fusions is a hallmark of senescence, consistent with molecular signs of senescence previously reported in term placentas. Telomere fusions result from telomere erosion but may occur independent of telomere length or telomerase activity (Cleal *et al.* 2018).

Human cytotrophoblasts from term placentas of uncomplicated pregnancies have exceptionally high rates of aneuploidy, while first- and second-trimester placentas have low and intermediate rates, respectively (Weier *et al.* 2010). Our findings suggest a mechanism driving placental aneuploidy. If senescence plays an important role in controlling the duration of pregnancy, and telomeres provide the timer, aneuploidy must be the inevitable outcome of this process. Why telomere–telomere fusions and aneuploidy, so ubiquitous in term placentas, do not promote malignancy as they do in somatic cells remains an enigma.

Human ESCs, derived from the blastocyst inner cell mass 5–6 days post-fertilization, divide indefinitely while maintaining genomic stability (Thomson *et al.* 1998). Ours is the first study to show that even late passage hESCs do not exhibit telomere–telomere fusions to the extent observed in the placenta. We cannot rule out that telomere–telomere fusions, below the limit of detection of our assay, may take place at later passages. But for the purpose of our study, hESCs provided an optimal negative control.

In conclusion, our study is the first to show telomere–telomere fusions in term placentas, in contrast to hESCs that escape senescence by virtue of their stemness.

## Declaration of interest

The authors declare that there is no conflict of interest that could be perceived as prejudicing the impartiality of this research letter.

## Funding

Financial support was provided by Conselho Nacional de Desenvolvimento Científico e Tecnológico (CNPq, Brazil, Grant number 204747/2018-0 for F B K) and the Stanley H Kaplan Fund.

## Author contribution statement

F B K designed and conducted the experiments, interpreted the data, and wrote the manuscript. F W contributed to the experimental design, interpretation of data and writing of the manuscript. D L contributed to the experiments and writing of the manuscript. R M contributed to experimental samples and writing of the manuscript. D L K designed the study, interpreted the data and wrote the manuscript.
